# CYP1B1 Mediates Cigarette Smoke–Induced Lipid Accumulation in Alveolar Type 2 Cells

**DOI:** 10.1096/fj.202501439RR

**Published:** 2025-09-19

**Authors:** Yin Zhu, Siddhika Gamare, Francesca Polverino, Caroline A. Owen, Payaningal R. Somanath, Xiaoyun Wang, Duo Zhang

**Affiliations:** ^1^ Clinical and Experimental Therapeutics, College of Pharmacy University of Georgia Augusta Georgia USA; ^2^ Charlie Norwood VA Medical Center Augusta Georgia USA; ^3^ Baylor College of Medicine, Medicine Houston Texas USA; ^4^ Division of Pulmonary and Critical Care Medicine Brigham and Women's Hospital, Harvard Medical School Boston Massachusetts USA; ^5^ Department of Medicine, Medical College of Georgia Augusta University Augusta Georgia USA

**Keywords:** COPD, e‐cigarettes, lipid metabolism, lung epithelial cell, oxidative stress

## Abstract

Alterations in lipid profiles have been shown in patients with chronic obstructive pulmonary disease (COPD), but the underlying molecular mechanisms remain unclear. In this study, we aimed to investigate the role of cytochrome P450 family‐1 subfamily B member 1 (CYP1B1) in cigarette smoke (CS)‐induced lipid accumulation in alveolar type II epithelial (AT2) cells. We observed a steady increase in CYP1B1 protein levels in AT2 cells from COPD patients. Additionally, CS exposure induced CYP1B1 expression in AT2 cells of murine lungs. In vitro, treatment with cigarette smoke extract (CSE) not only upregulated CYP1B1 expression but also triggered lipid accumulation in AT2‐like cells. Functionally, overexpression of CYP1B1 promoted lipid accumulation in A549 and MLE‐12 cells. Consistently, siRNA‐mediated CYP1B1 inhibition significantly reduced CSE‐induced lipid accumulation in AT2‐like cells. Furthermore, treatment with 2,3′,4,5′‐tetramethoxystilbene (TMS), a selective CYP1B1 inhibitor, reduced CSE‐induced lipid accumulation. TMS also attenuated CSE‐induced mitochondrial reactive oxygen species production and cell apoptosis. Taken together, our findings suggest that CYP1B1 is upregulated by CS exposure and plays a key role in CS‐induced lipid accumulation in AT2 cells. Targeting CYP1B1 may offer a potential therapeutic strategy for addressing lipid dysregulation and lung pathology in patients with COPD.

## Graphical abstract

Scheme of cigarette smoke (CS)‐induced lipid accumulation in alveolar type 2 (AT2) cells. Cigarette smoke (CS) exposure induces the expression of cytochrome P450 family 1 subfamily B member 1 (CYP1B1) by disrupting lipid metabolism in AT2 cells. The upregulation of CYP1B1 contributes to lipid accumulation within AT2 cells, leading to elevated oxidative stress and increased cellular apoptosis. Functionally, the inhibition of CYP1B1 using 2,3′,4,5′‐tetramethoxystilbene (TMS), a CYP1B1 inhibitor, effectively attenuates CS‐induced lipid accumulation in AT2 cells.

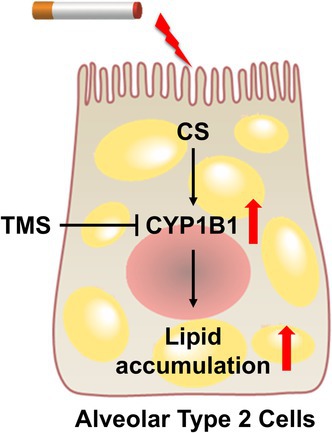

## 1 Introduction

Chronic obstructive pulmonary disease (COPD) is one of the leading causes of death worldwide. The annual medical cost of COPD treatment is expected to increase by more than $50 billion by 2029 [1, 2]. Despite the significant clinical and economic burden, the underlying molecular mechanisms of COPD remain poorly understood, and no pharmacological therapies are currently available to reverse disease‐associated damage. COPD is characterized by persistent airflow limitation caused by airway remodeling and emphysema, along with chronic pulmonary inflammation [3]. Cigarette smoke (CS) is identified as the major environmental risk factor for COPD development [4], as inhaled CS promotes pulmonary inflammation and disrupts alveolar epithelial homeostasis [5]. The alveolar epithelium plays a critical role in pulmonary gas exchange. Within this process, alveolar epithelial type I (AT1) cells exhibit greater susceptibility to external stimuli and lung injury compared to alveolar epithelial type II (AT2) cells [6, 7]. In response to lung injury, AT2 cells function as progenitor cells to facilitate tissue repair and restore normal lung function [6]. Additionally, AT2 cells release surfactant proteins into the alveoli by exocytosis of the secreting lamellar bodies, and contribute to surfactant protein recycling and degradation [8]. Exposure to CS has been reported to cause damage to alveolar epithelial cells and impair surfactant protein synthesis; however, the underlying mechanisms remain to be elucidated [8, 9, 10, 11].

CS exposure has been shown to disrupt pulmonary lipid metabolism through multiple cellular pathways [12]. Our previous study showed that cigarette smoke extract (CSE) induces lipid‐laden macrophage formation by upregulating cytochrome P450 family‐1 subfamily B member 1 (CYP1B1) [3]. Studies have reported that the enzymes belonging to the cytochrome P450 superfamily contribute to lipid metabolism, as CYP1B1 metabolizes arachidonic acid, the primary source of fatty acids [13]. In addition, *Cyp1b1* deficiency has been shown to protect mice from high‐fat diet–induced obesity, thereby further underscoring its critical role in the regulation of lipid metabolism [14]. Hence, we hypothesize that CS exposure induces CYP1B1 expression, thereby altering lipid metabolism and triggering lipid accumulation in AT2 cells. In this study, we further investigated the potential relationship between the role of CYP1B1 and CS‐induced lipid metabolic dysfunction in AT2 cells. Our findings showed that CYP1B1 expression was significantly increased in AT2 cells from patients with COPD compared to those from the nonsmoker control (NSC) group. Elevated CYP1B1 levels in AT2 cells were also confirmed in the lungs of mice following 6 months of CS exposure. When treated with CSE, the AT2‐like cell lines, A549 and MLE‐12, showed increased lipid accumulation. Functionally, overexpression of CYP1B1 through plasmid transfection promoted lipid accumulation in A549 cells. In contrast, the knockdown of CYP1B1 using siRNA reduced CSE‐induced lipid accumulation. In addition to CSE, e‐cigarette vapor extract (EVE) exposure also induced lipid accumulation, which was suppressed by CYP1B1 siRNA transfection in A549 cells. A selective CYP1B1 inhibitor, 2,3′,4,5′‐tetramethoxystilbene (TMS) [15, 16], effectively attenuated CSE‐induced lipid accumulation, mitochondrial reactive oxygen species (ROS) production, and cell apoptosis in AT2‐like cells. In summary, our study indicates that CYP1B1 is upregulated in AT2 cells from patients with COPD and CS‐exposed mice, and by CS exposure in AT2 cells in vitro. Additionally, CYP1B1 promotes the disruption of lipid metabolism, potentially leading to pathological lipid accumulation and contributing to the development of COPD.

## 2 Materials and Methods

### 2.1 Human Specimens and Ethics Approval

In this study, de‐identified human samples were used. Human sample research was approved and deemed to be nonhuman subject research by the institutional review board at Augusta University (IRB reference number: 2070085–1) and Brigham and Women's Hospital (IRB reference number: 1889310–1). The double immunofluorescence staining of CYP1B1 and SP‐C was performed in lung sections, and demographic and clinical characteristics are shown in Table 1. The lung sections in this cohort were provided by the Lung Tissue Research Consortium (LTRC) or the Department of Pathology at Brigham and Women's Hospital [17]. A power analysis was conducted to estimate the appropriate sample size. Based on mRNA expression data from the GSE37768 dataset, the mean mRNA level of *CYP1B1* was 924.2 ± 65.4 in the COPD group and 373.1 ± 79.2 in the non‐COPD group, indicating a large effect size (Cohen's d ≈7.59). Using a two‐sided *t*‐test with α = 0.05 and 80% power, the calculated minimum sample size is fewer than two participants per group. To ensure statistical rigor and account for biological variability and potential outliers, we included five samples per group.

**TABLE 1:** Lung immunostaining cohort: Demographic and clinical characteristics.

Characteristics	Nonsmokers^a^ (*N* = 5)	COPD patients^b^ (*N* = 5)	*p* ^c^
Number of males (%)	2 (40)	2 (40)	*p* > 0.999
Age (years)	54.0 ± 8.1	58.4 ± 7.5	*p* = 0.398
Pack‐years of smoking	0	47.6 ± 21.8	*p* = 0.001^d^
Number of current smokers (%)^e^	0 (0)	0 (0)	*p* > 0.999
FEV1 (% of predicted)	108.4 ± 20.6	26.6 ± 13.0	*p* < 0.001^f^
FEV1/FVC (% of predicted)	81 (76–96)	37 (31.5–56.5)	*p* = 0.009^g^

*Note:* The table shows the demographic and clinical characteristics of the patients with COPD and nonsmoker controls who underwent either a lung biopsy, lung volume reduction surgery, or lung transplantation. Data are presented as median (interquartile range) for data that were not normally distributed or mean ± SD for data that were normally distributed.

^a^ Nonsmokers were defined as individuals who had never smoked.

^b^ All COPD patients had forced expiratory volume in 1 s/forced vital capacity ratio (FEV1/FVC) < 0.7 whereas nonsmoker controls had FEV1/FCV > 0.7.

^c^ Categorical variables were analyzed with Chi‐square test. Statistical analyses included one‐way ANOVA tests for continuous variables (age, FEV1% predicted, FEV1/FCV, and pack‐years of smoking history) followed by pair‐wise comparisons using two‐tailed Student's *t*‐tests for data that were normally distributed or Mann–Whitney U tests for that were not normally distributed.

^d^ The pack‐years of smoking histories of the COPD patients were significantly different from those of the nonsmoker group by design (*p* = 0.001).

^e^ Current smokers were defined as active smokers at the time the samples were obtained or had stopped smoking < 1 year before the samples were obtained.

^f^ The FEV1 values for the COPD patients were significantly lower than those of the nonsmokers by design (*p* < 0.001).

^g^ The FEV1/FVC ratios for the COPD patients were significantly lower than those of the nonsmoker groups by design (*p* = 0.009).

### 2.2 Animal Studies

CS exposure was performed as described previously [17]. Wild‐type C57BL/6 mice that were sex‐ and age‐matched (8–10 weeks old) were purchased from Jackson Laboratory. All animal studies were approved by the Institutional Animal Care and Use Committees of Brigham and Women's Hospital and Charlie Norwood Veterans Affairs Medical Center, and were conducted in accordance with ARRIVE guidelines. Briefly, wild‐type mice in the CS group were exposed to mixed mainstream and side‐stream CS from 3R4F research cigarettes (University of Kentucky) for 2 h per day and 6 days per week in smoke‐exposure chambers (Teague Enterprises, https://teague‐ent.com), whereas the room air control group was exposed only to room air. Lungs were inflated, removed, fixed in formalin, and then embedded in paraffin for future analysis. Oxidized low‐density lipoprotein (OxLDL; 100 μg) (Athens Research & Technology, Athens, GA, USA) was intratracheally administered to mice on Day 0 in 100 μL sterile PBS. TMS (0.5 or 1.5 mg/kg, MedChemExpress) was administered to mice intraperitoneally on Days 1, 3, and 5 [18]. PBS served as the control. Mice were euthanized with 5% isoflurane, and lung sections were isolated on Day 7 post‐OxLDL treatment for experimental use.

### 2.3 Cell Culture, siRNA, and Plasmid Transfection

Human A549 (Catalog# CCL‐185) and murine MLE‐12 (Catalog# CRL‐2110) epithelial cell lines were purchased from American Type Culture Collection (ATCC, Manassas, VA, USA); these epithelial lines were selected for study as the epithelium is the initial site of lung injury following inhalation of CS or e‐cigarette vapor. The cells were cultured in DMEM complete medium (Thermo Fisher Scientific) containing 10% FBS (MilliporeSigma) and 1% Penicillin–Streptomycin (Thermo Fisher Scientific). All cells were cultured at 37°C in a humidified atmosphere with 5% CO_2_–95% air. Commercial siRNA of CYP1B1 (siCYP1B1, Catalog # AM16708, Assay ID 145515 or s64677) and siRNA control (siCon) were purchased from Thermo Fisher Scientific. The CYP1B1 plasmid was designed and subcloned into pBABE (Addgene, MA, USA). pBABE was a gift from David Ron (Addgene plasmid # 21836). Lipofectamine 2000 (Thermo Fisher Scientific) was used for transfection according to the manufacturer's protocol.

### 2.4 CSE, E‐Cigarette Vapor Extract (EVE), and 2,3′,4,5′‐Tetramethoxystilbene (TMS) Treatment

CSE was prepared as described before [3]. Briefly, a lit research cigarette (3R4F, University of Kentucky) was used to bubble smoke into 10 mL of DMEM, creating a 100% CSE solution. This freshly prepared CSE solution was then filtered and diluted to achieve the specific concentrations required for each experiment. E‐cigarettes (Juicy Bar JB5000 Disposable Vape‐clear flavor) were purchased from Jvapes (Prescott, AZ, USA). The e‐cigarette liquid has a 13 mL capacity with 5000 puff counts and a rechargeable battery. The product contains a 1.2 Ohm Mesh Coil and a nicotine strength of 50 mg/mL (5%). EVE was prepared as described before [19]. Briefly, negative pressure from a syringe was applied to the activated e‐cigarette. After the e‐cigarette vapor was exhaled, this procedure was repeated for a total of 30 puffs. This process produced 10 mL of medium, which constituted a 100% EVE stock solution. The 100% EVE stock was then diluted to the desired concentration for the experiment. CSE and EVE concentrations ranging from 5% to 15% were added to the culture medium and were freshly prepared for each experiment. TMS from MedChemExpress was dissolved in PBS and aliquoted as a 10 mM stock from which 10 μM TMS was added to the culture medium 2 h before the CSE treatment.

### 2.5 Immunofluorescence Staining

Immunofluorescence staining was performed as previously described [20]. Lung sections from nonsmoker controls or patients with COPD and mice exposed to CS or room air for 6 months were incubated with anti‐CYP1B1 antibody (PA5‐95277, rabbit polyclonal IgG, Thermo Fisher Scientific, 1:250) and anti‐SP‐C antibody (sc‐7706, goat polyclonal IgG, Santa Cruz, 1:100) overnight at 4°C. The sections were washed with PBS and incubated with a secondary antibody conjugated to Alexa Fluor 488 (A32790, donkey polyclonal IgG, Thermo Fisher Scientific) or 555 (A‐21432, donkey polyclonal IgG, Thermo Fisher Scientific). A549 or MLE‐12 epithelial cell lines were incubated with 10% CSE for 3 days and fixed with formaldehyde solution (Millipore Sigma). The cells were washed with PBS and incubated with anti‐CYP1B1 antibody (PA5‐95277, 1:500) overnight. Following the incubation of a secondary antibody conjugated to Alexa Fluor 546 (A‐11071, goat polyclonal IgG), the DAPI‐containing mounting medium (ab104139, Abcam) was used for nucleus staining and mounting. Imaging was captured by Carl Zeiss Observer Z1 microscope, and the fluorescence intensity was quantified using ImageJ.

### 2.6 Reactive Oxygen Species (ROS) Detection

Cells were cultured with 5 μM MitoSOX Red dye (Thermo Fisher Scientific) for 10 min to determine mitochondrial oxidative stress. After incubation, cells were washed with PBS three times and then fixed with a 4% formaldehyde solution (MilliporeSigma) for 1 h before imaging. The nuclei of the cells were stained using 4′,6‐diamidino‐2‐phenylindole (DAPI, Abcam, Waltham, MA, USA). Images were taken using a Carl Zeiss Observer Z1 microscope (Jena, Germany). The fluorescence intensity of these images was quantified using ImageJ software (U.S. National Institutes of Health, Bethesda, MD, USA).

### 2.7 Oil Red O (ORO) Staining

ORO staining was performed as described previously [21]. One hundred percent isopropanol‐saturated ORO (MilliporeSigma) was freshly prepared as a stock solution. The ORO working solution was prepared by mixing 6 mL of stock solution with 4 mL of ddH_2_O. Fixed cells were washed with ddH_2_O three times and stained at room temperature for 30 min. After staining, the cells were washed with ddH_2_O, and images were taken using the Carl Zeiss Observer Z1 microscope. The overall lipid accumulation was quantified by eluting ORO dye by adding 100% isopropanol and incubating for 10 min with gentle shaking. The ORO values were measured at 500 nm using a plate reader, with 100% isopropanol as the blank.

### 2.8 Annexin V Staining

Cellular apoptosis was measured using the Annexin V‐FITC detection kit (Abcam) following the manufacturer's protocol. The cells were seeded and treated with the desired conditions and incubated with the Annexin V‐FITC reagent in the dark for 5–10 min. The cells were then washed with PBS and fixed in 2% formaldehyde for visualization. The images were captured by a Carl Zeiss Observer Z1 microscope. The fluorescence intensity from the cells in each group was quantified using ImageJ software.

### 2.9 Cell Viability Assay

Cell viability was determined using the CellTiter 96 AQ_ueous_ One Solution Cell Proliferation Assay kit (Promega). Briefly, the CellTiter reagent was added to the treated cells at a final concentration of 10%. The cells were then incubated at 37°C for 2 h in a humidified, 5% CO_2_ atmosphere. The absorbance was measured using a plate reader at 490 nm.

### 2.10 LipidTOX Staining

LipidTOX staining was performed as previously described [21]. Fixed cells were washed with ddH_2_O 3 times and incubated with LipidTOX dye (Thermo Fisher Scientific) for 30 min. DAPI mounting solution was used to stain the nucleus. For frozen murine lung sections, anti‐SP‐C antibody (sc‐7706, 1:100) was incubated overnight at 4°C on the lung sections. The sections were washed with PBS and incubated with secondary antibody conjugated to Alexa Fluor 488 (A‐11055, donkey polyclonal IgG, Thermo Fisher Scientific). The mounting medium containing DAPI (ab104139, Abcam) was used for mounting and staining the nuclei.

### 2.11 Western Blot Analyses

Western blot experiments were performed as previously described [22]. Cells were collected and lysed with RIPA buffer (RPI Research Products, Mount Prospect, IL, USA) with the addition of a protease inhibitor (Thermo Fisher Scientific). The protein lysates were first separated using SDS‐PAGE gels and then transferred onto PVDF membranes (MilliporeSigma). The PVDF membranes were blocked and incubated with a primary polyclonal CYP1B1 antibody (PA5‐95277, Thermo Fisher Scientific) overnight. The membrane was subsequently washed with TBST solution and incubated with a secondary anti‐rabbit HRP‐conjugated antibody (HAF008, R&D Systems) for 1 h. To detect β‐actin, the membrane was stripped with stripping buffer (Thermo Fisher Scientific) and incubated overnight with a primary monoclonal anti‐*β*‐actin antibody (A5441, Millipore Sigma). This was followed by incubation with an anti‐mouse HRP‐conjugated antibody (HAF007, R&D Systems). Images were taken using the ChemiDoc imaging system (Bio‐Rad, Hercules, CA, USA). ImageJ software was used to quantify the intensity of the bands.

### 2.12 RNA Preparation, Reverse Transcription, RT‐qPCR, and PCR Array

Total RNA was extracted using the RNAqueous Total RNA Isolation Kit (Thermo Fisher Scientific). cDNA was synthesized using the high‐capacity cDNA reverse transcription kit (Thermo Fisher Scientific) following the manufacturer's instructions. Human and mouse TBP were used as housekeeping genes. Primers used in the study were purchased from Integrated DNA Technologies (Coralville, IA, USA). PowerUP Green Master Mix (Thermo Fisher Scientific) was used for RT‐qPCR. RT‐qPCR was performed and analyzed using the QuantStudio 3 Real‐Time PCR system (Thermo Fisher Scientific). The RT^2^ Profiler PCR Array for Mouse Extracellular Matrix and Adhesion Molecules was performed following the protocol from the manufacturer (Qiagen, Germantown, MD, USA). The primer sequences are listed in Table 2.

**TABLE 2:** Primers used in RT‐qPCR.

Gene symbol	Forward (5′‐3′)	Reverse (5′‐3′)
Human TBP	GATAAGAGAGCCACGAACCAC	CAAGAACTTAGCTGGAAAACC
Human CYP1B1	GCCACTATCACTGACATCTTCGG	CACGACCTGATCCAATTCTGCC
Human FASN	TTCTACGGCTCCACGCTCTTCC	GAAGAGTCTTCGTCAGCCAGGA
Human SCD1	CCTGGTTTCACTTGGAGCTGTG	TGTGGTGAAGTTGATGTGCCAGC
Human LXR‐β	CTTCGCTAAGCAAGTGCCTGGT	CACTCTGTCTCGTGGTTGTAGC
Mouse Tbp	TCAAACCCAGAATTGTTCTCC	GGGGTAGATGTTTTCAAATGC
Mouse Ccl1	GCTTACGGTCTCCAATAGCTGC	GCTTTCTCTACCTTTGTTCAGCC
Mouse Il‐6	TAGTCCTTCCTACCCCAATTTCC	TTGGTCCTTAGCCACTCCTTC
Mouse Tnf‐α	GGTGCCTATGTCTCAGCCTCTT	GCCATAGAACTGATGAGAGGGAG

### 2.13 Bioinformatics Methods for CYP1B1 Expression Analysis

The mRNA expression of *CYP1B1* in the lungs of nonsmokers, smokers, and COPD patients (GSE37768; entitled “Expression data in lung tissue from moderate COPD patients, healthy smokers and nonsmokers”); and in AT2 cells from participants with versus without COPD (GSE29133; entitled “Transcriptome in alveolar epithelial type II cells isolated from normal and COPD lungs of adult human”) was obtained from the Gene Expression Omnibus (GEO) database. *CYP1B1* expression data were acquired and analyzed using GEO2R. GraphPad Prism software (Boston, MA, USA) was used for graphically representing values from the samples.

### 2.14 Statistics Analysis

All data in this study were presented as means ± SD and were analyzed with SigmaPlot (Systat Software). The difference between the two experimental groups was analyzed using a two‐tailed unpaired Student's *t*‐test for variables that fall in the normal distribution, while data that were not normally distributed were analyzed using Mann–Whitney U tests. Differences between multiple (three or more) experimental groups were compared using one‐way ANOVAs with Tukey's tests for data with a normal distribution; Mann–Whitney U tests were used for analyzing data that were not normally distributed. Groups were considered to be statistically significantly different when *p* ≤ 0.05.

## 3 Results

### 3.1 CYP1B1 Is Upregulated in AT2 Cells From COPD Lungs

**FIGURE 1:**
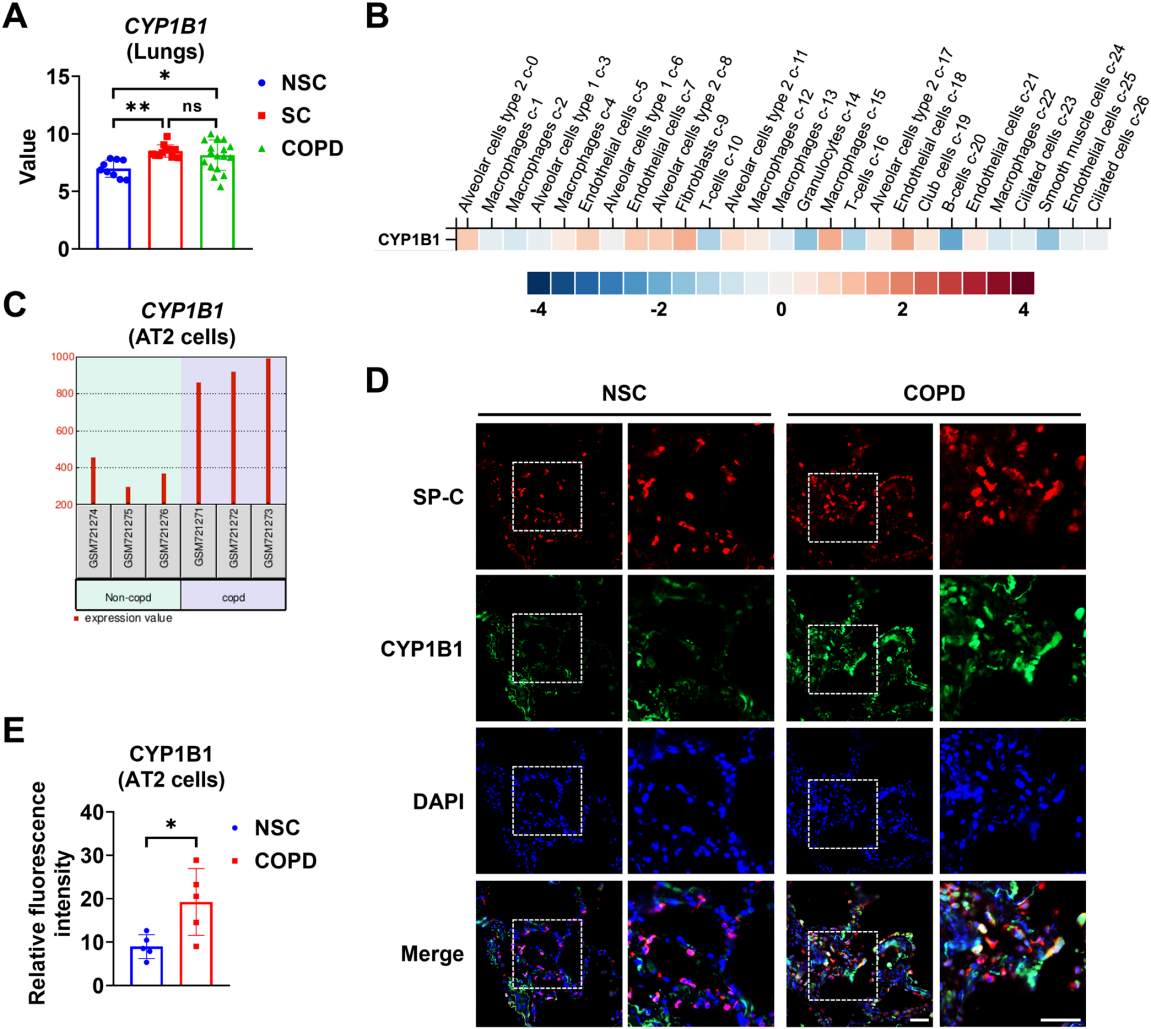
CYP1B1 is upregulated in AT2 cells from COPD lungs. (A) *CYP1B1* sample values in lungs from nonsmokers (NSC) group (*n* = 9), smokers (SC) group (*n* = 11), and COPD group (*n* = 18) from the NCBI GEO database (GEO accession: GSE37768). (B) The lung single‐cell RNA‐sequencing data provided by the Human Protein Atlas database show the relative *CYP1B1* mRNA expression in various human lung cells (accessed on 03/28/2025). (C) *CYP1B1* sample values in AT2 cells from non‐COPD controls (*n* = 3) and COPD patients (*n* = 3) from NCBI GEO database (GEO accession: GSE29133). (D and E) Antibodies against CYP1B1 and SP‐C were used to conduct double immunofluorescence staining in lung sections of nonsmoker control subjects (*n* = 5) and patients with COPD (*n* = 5). Representative images of immunostained lung sections are shown in D; the relative fluorescence intensity of CYP1B1 in SP‐C positive cells was quantified (E). Results are presented as means ± SD and are from three independent experiments. *p < 0.05.

To explore whether *CYP1B1* expression is dysregulated in COPD, we accessed the GEO database (GSE37768), where the authors compared gene profiles in lung tissue samples from patients with moderate COPD, smokers (SC), and nonsmokers (NSC) [23]. Among all the upregulated genes, *CYP1B1* mRNA expression showed a significant increase in the lungs of smokers and COPD patients compared to nonsmokers (Figure 1A). Single‐cell RNA Sequencing (sc‐RNA seq) analysis from the Human Protein Atlas database indicates that AT2 cells, fibroblasts, macrophages, and endothelial cells exhibit high *CYP1B1* mRNA levels (Figure 1B) [24]. We further accessed another GEO database (GSE29133) and found that the *CYP1B1* expression level was significantly higher in AT2 cells from COPD patients compared with those from the non‐COPD controls (Figure 1C). To confirm this finding, we conducted double immunofluorescence staining of sections of the lung from age‐ and sex‐matched former smokers with COPD and nonsmoker controls without COPD. The demographic and clinical data for this cohort are shown in Table 1. We observed a significant increase in CYP1B1 protein in AT2 cells (SP‐C positive cells) from COPD patients compared to nonsmoker controls (Figure 1D,E).

### 3.2 CS Exposure Induces CYP1B1 Expression in AT2 Cells

**FIGURE 2:**
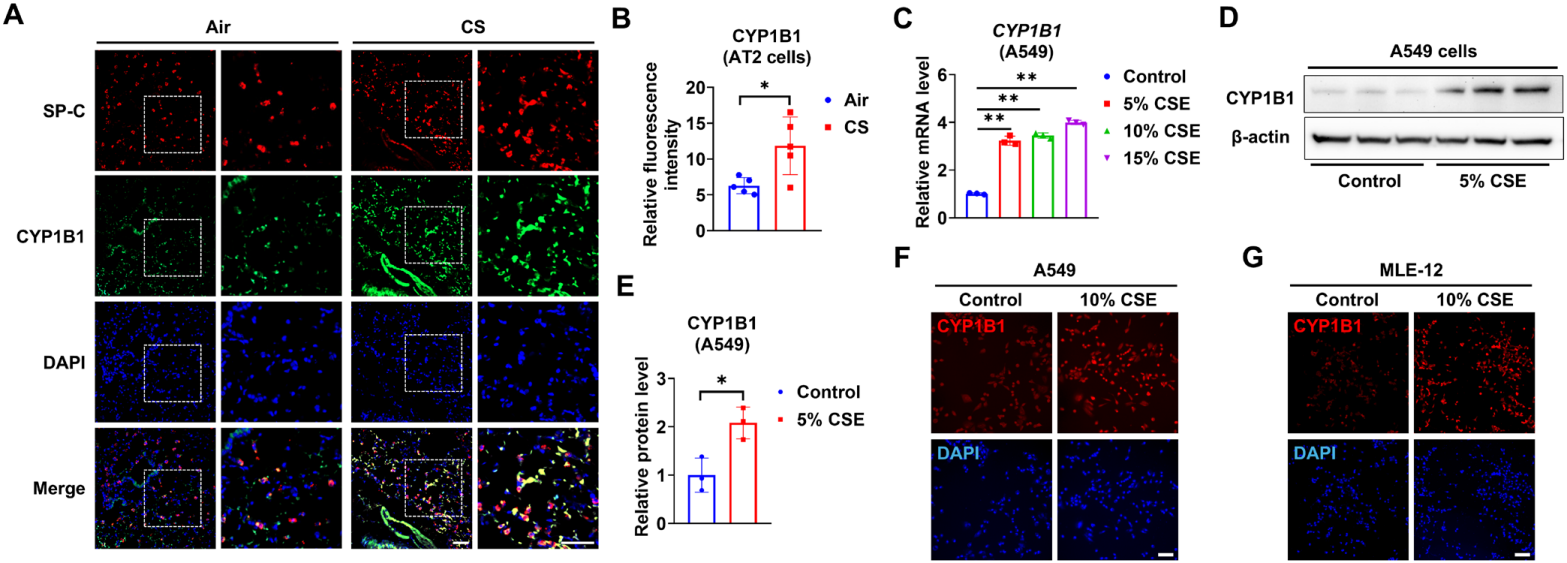
CS exposure induces CYP1B1 expression in AT2 cells. (A) Antibodies against CYP1B1 and SP‐C were used to conduct double immunofluorescence staining in lung sections of mice exposed to CS or room air for 6 months (*n* = 5/group); the relative fluorescence intensity of CYP1B1 in SP‐C‐positive cells was quantified (B). Scale bar = 50 μm. (C) A549 cells were incubated with varying concentrations of CSE as indicated for 24 h. *CYP1B1* mRNA levels were quantified by RT‐qPCR. (D and E) A549 cells were incubated with or without 5% CSE for 3 days. CYP1B1 protein levels (*n* = 3 per group) were determined by western blotting (D) and quantified (E). (F and G) A549 and MLE‐12 were treated with or without 10% CSE for 3 days. An antibody against CYP1B1 was used to perform immunofluorescence staining. Scale bar = 100 μm. DAPI was used for nucleus staining. Results are presented as means ± SD and are from three independent experiments. *p < 0.05, **p < 0.01.

We next measured CYP1B1 protein expression using the lung sections of the mice exposed to CS or room air. Immunostaining for CYP1B1 was significantly increased in SP‐C‐positive AT2 cells from those mice exposed to CS for 6 months compared with those from the air control group (Figure 2A,B). CS‐induced increases in *CYP1B1* mRNA levels were confirmed in human A549 cells exposed to different concentrations of CSE (Figure 2C). Consistent with this result, CSE exposure increased CYP1B1 protein levels in A549 cells, as shown by western blotting (Figure 2D,E) and immunofluorescence staining (Figure 2F). A similar result was observed in a murine AT2‐like cell line, MLE‐12, following CSE incubation (Figure 2G).

### 3.3 CS Exposure Induces Lipid Accumulation in AT2‐Like Cells

**FIGURE 3:**
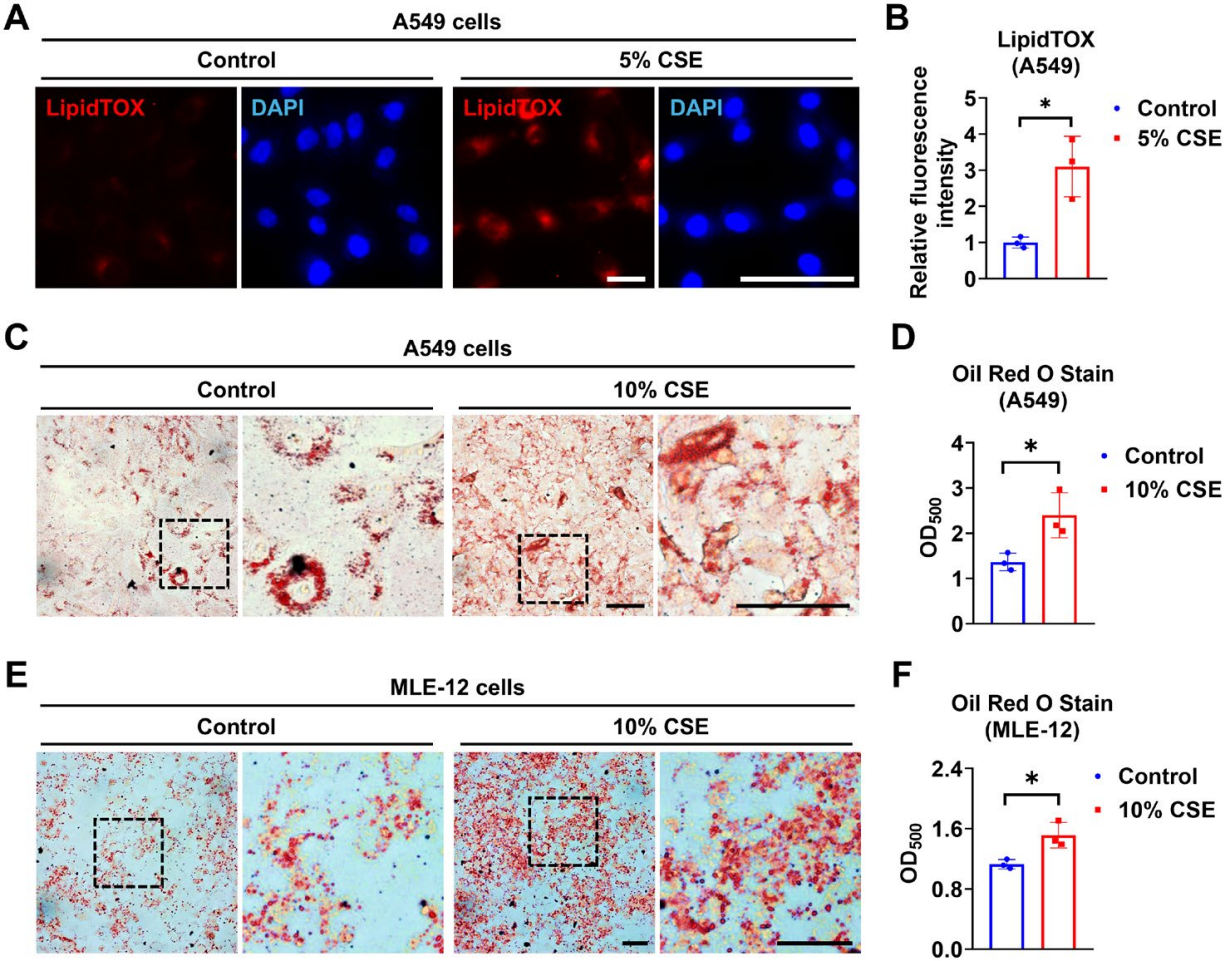
CS exposure induces lipid accumulation in AT2‐like cells. (A and B) A549 cells were incubated with or without 5% CSE for 3 days. The intracellular lipid droplets were stained using LipidTOX dye, and the nucleus was stained by DAPI (A). Relative fluorescence intensity was quantified (B). (C–F) A549 and MLE‐12 cells were treated with or without 10% CSE for 3 days. Lipid droplets were stained and quantified using ORO (C and D) in A549 cells. Lipid droplets were stained (E) and quantified (F) in MLE‐12 cells. Results are presented as means ± SD and are from three independent experiments. Scale bar = 50 μm. *p < 0.05.

To understand the effect of CS on lipid metabolism in AT2 cells, we conducted LipidTOX staining and found that CSE incubation promotes lipid accumulation in A549 cells (Figure 3A,B). Similarly, after incubating with 10% CSE for 3 days, both A549 and MLE‐12 cells showed more lipid accumulation than the untreated group (Figure 3C–F) as assessed by Oil Red O (ORO) staining. There is growing interest in understanding how e‐cigarette use contributes to lung injuries, including e‐cigarette or vaping product use‐associated lung injury (EVALI) [25, 26]. Our team has shown that lipid accumulation increases in macrophages after EVE exposure [3]. Here we show that, when treated with 10% EVE for 3 days, A549 cells had increased lipid droplet accumulation (Figure E1A,B). In addition, both CSE and EVE induced lipid accumulation in a concentration‐dependent manner (Figure E1C,D). These findings indicate that CSE and EVE treatment induce lipid accumulation in AT2‐like cells.

### 3.4 CYP1B1 Regulates CS‐Induced Lipid Accumulation in AT2‐Like Cells

**FIGURE 4:**
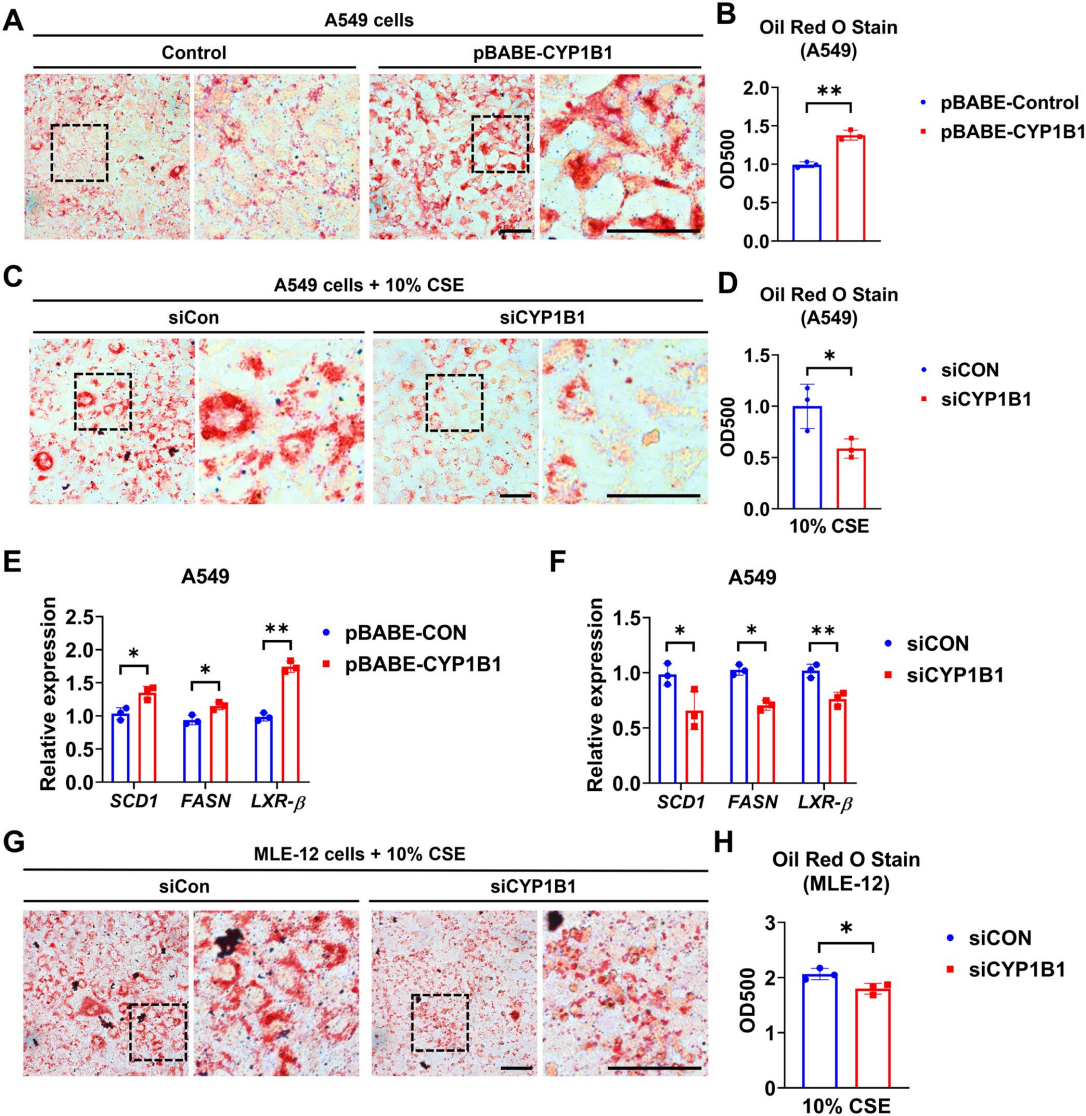
CYP1B1 regulates CS‐induced lipid accumulation in AT2‐like cells. A549 cells were transfected with pBABE‐puro or pBABE‐CYP1B1 and cultured for 3 days. The lipid accumulation in the cells was determined by ORO staining (A) and quantified (B). (C and D) A549 cells were transfected with siCon or siCYP1B1 and incubated with 10% CSE for 3 days. ORO was used to determine and quantify the lipid accumulation in A549. Scale bar = 50 μm. A549 cells were transfected with pBABE‐puro and pBABE‐CYP1B1 or siCon and siCYP1B1, and then cultured for 3 days. (E and F) mRNA levels of *SCD1*, *FASN*, and *LXR‐β* were confirmed with RT‐qPCR. MLE‐12 cells were transfected with siCon or siCYP1B1 and incubated with 10% CSE for 3 days. Lipid accumulation was confirmed by ORO staining (G) and quantified (H). Results are presented as means ± SD and are from three independent experiments. *p < 0.05, **p < 0.01.

We next overexpressed CYP1B1 in A549 cells and found that CYP1B1 overexpression significantly enhanced intracellular lipid accumulation (Figure 4A,B). In contrast, CYP1B1 knockdown markedly suppressed CSE‐induced lipid accumulation (Figure 4C,D). Overexpression of CYP1B1 upregulated key genes involved in lipid metabolism, including Stearoyl‐CoA Desaturase 1 (SCD1), Fatty Acid Synthase (FASN), and Liver X Receptor Beta (LXR‐*β*), whereas their expression was reduced upon CYP1B1 knockdown (Figure 4E,F). Similarly, CYP1B1 knockdown reduced CSE‐induced lipid accumulation in mouse MLE‐12 cells (Figure 4G,H) and attenuated EVE‐induced lipid accumulation in A549 cells (Figure E2A,B). These findings suggest that CYP1B1 plays a role in lipid accumulation in AT2‐like cells.

### 3.5 TMS Suppresses CSE‐Induced Lipid Accumulation, ROS Generation, and Apoptosis in AT2‐Like Cells

**FIGURE 5:**
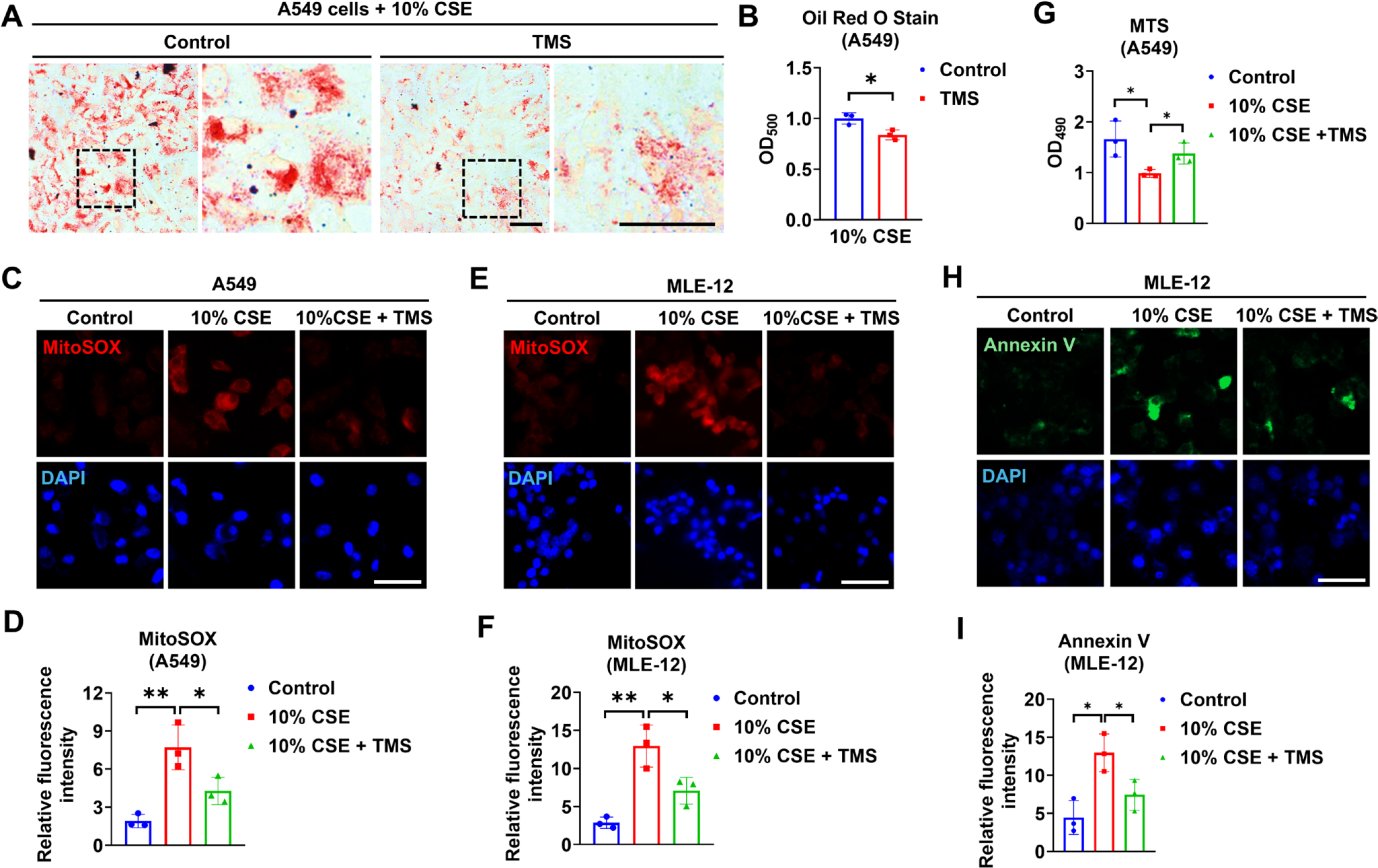
TMS suppresses CSE‐induced lipid accumulation, ROS generation, and apoptosis in AT2‐like cells. A549 or MLE‐12 cells were preincubated with 10 μM TMS for 2 h, and then treated with or without 10% CSE (A and B). CSE was added once and incubated with the cells for 3 days. The lipid accumulation was determined by ORO staining and quantified. MitoSOX dye was used to determine the mitochondrial ROS level for A549 (C) and mitochondrial ROS were quantified (D). MitoSOX dye was added to MLE‐12 cells (E) and mitochondrial ROS were quantified (F). (G) CellTiter reagent at a final concentration of 10% was incubated with the treated A549 cells, and the absorbance at 490 nm was measured. Annexin V dye was added to the MLE‐12 cells at the end of treatment. The cells were then fixed for imaging (H), and quantification of annexin V staining was performed (I). Scale bar = 50 μm. Results are presented as means ± SD and are from three independent experiments. *p < 0.05, **p < 0.01.

TMS is a selective CYP1B1 inhibitor [15, 16]. We tested its effect on lipid accumulation and readouts of epithelial injury (increased mitochondrial oxidative stress and apoptosis) in CSE‐treated AT2‐like cells. In line with the findings above, TMS significantly suppressed the CSE‐induced lipid accumulation (Figure 5A,B). In addition, TMS treatment inhibited CSE‐induced mitochondrial oxidative stress in A549 and MLE‐12 cells (Figure 5C–F). TMS also attenuated the cell apoptosis caused by CSE exposure, as demonstrated by the CellTiter assay (Figure 5G) and Annexin V staining (Figure 5H,I).

### 3.6 TMS Attenuates Lipid Accumulation in AT2 Cells and Inflammation In Vivo

**FIGURE 6:**
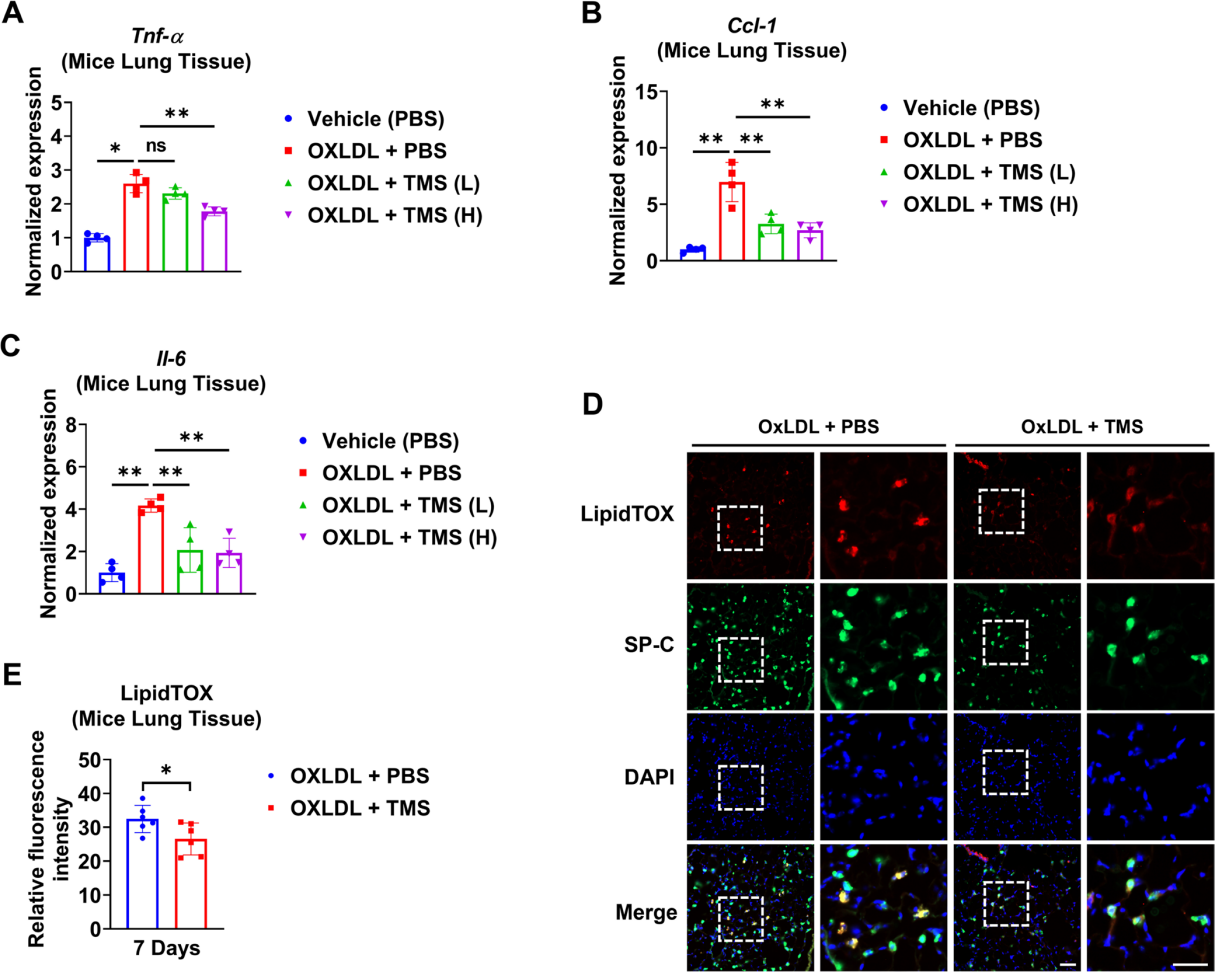
TMS inhibits lipid accumulation in AT2 cells and inflammatory response in murine lungs. OxLDL (100 μg) was intratracheally administered to mice on Day 0. Low‐dose (0.5 mg/kg, L) or high‐dose (1.5 mg/kg, H) TMS or PBS (control) was administered to mice intraperitoneally on Days 1, 3, and 5. Mice were euthanized, and lung sections were collected on Day 7 post‐OxLDL treatment. The expression of pro‐inflammatory markers Tnf‐α (A), Ccl‐1 (B), and Il‐6 (C) was measured using RT‐qPCR in murine lungs (*n* = 4/group). OxLDL (100 μg) was delivered by the intratracheal route to mice, and 1.5 mg/kg TMS (or PBS) was then delivered to mice by intraperitoneal injections on Days 1, 3, and 5. The murine lung sections (*n* = 6/group) were collected on Day 7. Immunostaining of lung sections with an antibody against SP‐C and LipidTOX dye was used to confirm lipid accumulation in AT2 cells. The relative fluorescence intensity of LipidTOX in SP‐C‐positive cells was measured (D) and quantified (E). Scale bar = 50 μm. ns *p* > 0.05, *p < 0.05, **p < 0.01.

Based on our in vitro findings, we next evaluated the effect of TMS in vivo. OxLDL treatment increased the expression of pro‐inflammatory cytokines in murine lungs, and this effect was significantly reversed by TMS in a dose‐dependent manner (Figure 6A–C). Furthermore, TMS effectively reduced OxLDL‐induced lipid accumulation in SP‐C positive AT2 cells compared with the PBS group (Figure 6D,E).

## 4 Discussion

COPD is a chronic inflammatory disease associated with pathological changes to lung structure and is the third leading cause of death worldwide [1]. Current therapeutic approaches for COPD include inhaled bronchodilators and corticosteroids, nebulized ensifentrine, and systemic dupilumab, all of which have been shown to reduce exacerbation rates while improving symptoms, pulmonary function, and overall quality of life [27, 28, 29, 30]. However, current therapies do not eliminate the high morbidity and mortality associated with COPD. The mechanisms underlying the pathogenesis of COPD have not been fully elucidated. The airway epithelium is the first tissue barrier to encounter external insults and noxious particles; therefore, airway epithelial cells are crucial for maintaining pulmonary homeostasis in the setting of lung injury [31]. Unlike AT1 cells, AT2 cells are the progenitor cells in the lung, as they can differentiate into AT1 cells during lung injury [32, 33]. In addition, AT2 cells play a central role in pulmonary metabolism and are key regulators of pulmonary surfactant, which is composed of approximately 90% lipids [34, 35]. A previous study demonstrated that CS exposure disrupts lipid metabolism in AT2 cells in mice, identifying fatty acid synthase as a key regulator of CS‐induced alterations in lipid biosynthesis within these cells [36]. However, whether other pathways contribute to CS‐induced intracellular lipid alterations in AT2 cells in the lungs has been unclear.

The role of cytochrome P450 (CYP) enzymes has been extensively studied in the context of metabolic diseases [37]. Among them, CYP1B1 is upregulated during adipogenesis, suggesting that it regulates metabolism [38]. CYP1B1 also participates in the metabolism of arachidonic acid, which is involved in fatty acid synthesis and is known to be expressed in lung tissue [39, 40]. Fatty acids are involved in the production of triacylglycerol and phospholipid, which are critical for forming lipid droplets [41, 42]. Previous research shows that CYP1B1 mediates xenobiotic metabolism and contributes to lung injury [37, 43]. Moreover, CYP1B1 expression has been detected in the lungs of both smokers and nonsmokers; however, its specific contribution to the pathogenesis of COPD remains unclear [44]. CS has been reported to alter cellular metabolic function by increasing inflammation and oxidative stress [45]. Our team has previously reported that CSE exposure can alter the metabolism of alveolar macrophages, ultimately leading to the formation of lipid‐laden macrophages via CYP1B1 [3]. CSE exposure impairs the function of macrophages in recycling lipids, resulting in excessive lipid accumulation in the lung, which imposes additional stress on the alveolar epithelium [46, 47]. Washino and colleagues have reported that temsirolimus treatment induces macrophage depletion and accumulation of surfactant lipids in an interstitial lung disease model, leading to alveolar epithelial injury and pulmonary inflammation [48]. However, it remains largely unclear whether CS exerts direct effects on lipid metabolism in AT2 cells and whether CYP1B1, a regulator of lipid metabolism in these cells, contributes to the pathogenesis of COPD. In this study, we report for the first time that the CYP1B1 protein level is significantly increased in AT2 cells in patients with COPD compared with nonsmoking controls. When exposed to CSE, CYP1B1 is induced in AT2‐like cells at both the mRNA and protein levels, accompanied by elevated intracellular lipid accumulation.

CYP1B1 is a member of the Cytochrome P450 enzyme family, which plays a key role in metabolizing endogenous compounds such as lipids, proteins, and hormones to maintain physiological homeostasis [49]. To investigate whether CS‐induced upregulation of CYP1B1 contributes to the associated increase in intracellular lipid accumulation in AT2‐like cells, we used complementary loss‐of‐function, gain‐of‐function, and CYP1B1 enzyme inhibition approaches in vitro. Using a loss‐of‐function study approach in which we knocked down CYP1B1 expression via siRNA transfection, we demonstrated a marked reduction in CSE‐induced lipid accumulation in AT2‐like cells. Similar results were obtained when cells were treated with a selective CYP1B1 inhibitor (TMS). Conversely, overexpressing CYP1B1 using plasmid transfection resulted in increased intracellular lipid accumulation in AT2‐like cells. Moreover, our findings demonstrate that CYP1B1 regulates the expression of SCD1, FASN, and LXR‐*β*, key genes involved in lipid metabolism. Furthermore, reductions in intracellular lipid accumulation induced by TMS‐induced inhibition of CYP1B1 were associated with reductions in readouts of cellular injury in AT2‐like cells (mitochondrial stress and apoptosis) when the cells were exposed to CSE. Based on our prior observations in macrophages and the current study's findings in epithelial cells, CYP1B1 is thus emerging as a promising target for regulating pathologic lipid accumulation in two key cellular culprits in the pathogenesis of COPD.

Another novel finding of our study is that treatment with EVE induced lipid accumulation in AT2‐like cells, a process that was markedly attenuated by silencing CYP1B1. Thus, CYP1B1 also contributes to EVE‐induced lipid accumulation in AT2‐like cells. It is noteworthy in this respect that the use of e‐cigarettes is rapidly increasing in teenagers and young adult populations, and e‐cigarettes have been advertised by their manufacturers as a less harmful alternative to cigarettes for smokers [25, 50]. However, there is increasing evidence that the usage of e‐cigarettes can be associated with different types of lung injury [51]. Clinical studies show that the use of e‐cigarettes increases the risk of airway epithelial cell injury [52, 53]. Sohal et al. reported that exposure to EVE induces toxicities in epithelial cells similar to those observed with CSE [54]. However, the relationship between these experimental doses and actual user exposure, as well as the underlying mechanisms of cellular injury, has been unclear. To address this gap, we evaluated EVE as an injury model to investigate whether EVE, like CS, induces lipid accumulation in AT2‐like cells. Our findings align with the existing hypothesis regarding the potentially harmful effect of EVE by showing that EVE induces intracellular lipid accumulation in AT2‐like cells and that this is mediated, in part, by CYP1B1.

Dysregulation of alveolar lipids has been reported in multiple pulmonary diseases, including COPD, idiopathic pulmonary fibrosis, and acute lung injury or acute respiratory distress syndrome [34]. Although most of the dysregulated lipid metabolism in the lungs was reported to be related to inflammation, knowledge gaps exist in the underlying mechanisms. Our data show that inhibiting CYP1B1 effectively inhibits the CS‐ and EVE‐induced lipid accumulation in AT2‐like cells. CSE is known to increase mitochondrial ROS levels in macrophages and bronchial epithelial cells [21, 55], which induce cellular senescence [56], and also promote apoptosis of alveolar septal cells [57]. In our study, CSE increased mitochondrial ROS levels and induced apoptosis in AT2‐like cells, and both of these processes were reduced by incubating the cells with the CYP1B1 inhibitor. Thus, our data indicate that CYP1B1 inhibition has therapeutic potential in COPD. Furthermore, *Cyp1b1* deficiency has been shown by other groups to attenuate high‐fat diet‐induced obesity and improve glucose tolerance. As type 2 diabetes mellitus is a common comorbidity in patients, these observations further support the notion that targeting CYP1B1 has therapeutic potential in COPD [14, 58].

Our study has several limitations. Although the sample size is limited (*n* = 3 per group) for both the control and COPD patient groups in the GEO dataset (GSE29133), our data show that CYP1B1 expression is significantly higher in AT2 cells in patients with COPD than in the control groups. The CYP superfamily mediates drug metabolism, and the CYP1, CYP2, and CYP3 families are primarily responsible for the oxidative metabolism of exogenous and endogenous compounds [59]. In the CYP1 family, human CYP1B1 exhibits approximately 40% homology with CYP1A1 and CYP1A2, along with shared metabolic activities [59]. Whether inhibition of CYP1B1 using TMS may lead to compensatory increases in the expression of CYP1A1 and CYP1A2 is not clear, but will be evaluated in future studies. Whether CS‐ and EVE‐induced lipid accumulation in AT2 cells has pathologic consequences beyond those evaluated in this study (including alterations in surfactant levels and function) is unclear and also needs to be evaluated in future studies. Additionally, the in vitro CSE model lacks certain components, such as insoluble tars, that are present in environmental smoke, and no established conversion factor exists to relate in vitro condensate concentrations to actual human airway doses [60]. Future studies will also evaluate whether treating CS‐exposed mice with TMS can reduce the progression of COPD‐like disease to further explore the potential of CYP1B1 inhibition as a therapeutic approach for COPD.

In conclusion, our data suggest that CYP1B1 is upregulated in AT2 cells in response to CS exposure. In addition, our study suggests that CYP1B1 plays a critical role in CS‐induced lipid accumulation in AT2‐like cells with deleterious functional consequences for these cells. Targeting CYP1B1 may offer a promising therapeutic strategy to mitigate CS‐induced lipid accumulation and potentially benefit patients with COPD.

## Author Contributions

**Y.Z.:** investigation, methodology, formal analysis, data curation, writing, review and editing, writing, original draft. **S.G.:** methodology, investigation, formal analysis, data curation. **F.P., C.A.O**., and **P.R.S**.: supervision, resources, writing, review and editing, conceptualization. **X.W**.: methodology, data curation, formal analysis, supervision, resources, writing, review and editing, funding acquisition. **D.Z.:** supervision, resources, writing, review and editing, conceptualization, funding acquisition, formal analysis, conceptualization.

## Conflicts of Interest

The authors declare that they have no known competing financial interests or personal relationships that could have appeared to influence the work reported in this paper. C.A.O. is currently an employee of AstraZeneca BioPharmaceuticals R&D but has no relevant conflicts of interest.

## Supporting information


**Figure E1.** EVE induces lipid accumulation in A549 cells. A549 cells were treated with 10% EVE for 3 days. Lipid droplets in A549 cells treated with 10% EVE were stained and quantified using ORO (A and B). (C) ORO staining was used to confirm the lipid accumulation after 3‐day CSE and EVE incubation (from 5%–15%). (D) The ORO staining was then quantified. Results are presented as means ± SD and are from three independent experiments. ns *p* > 0.05, * *p* < 0.05, ** *p* < 0.01.
**Figure E2**. Inhibiting CYP1B1 suppresses EVE‐induced lipid accumulation in A549 cells. A549 cells were transfected with siCon or siCYP1B1 and cultured for 3 days. The lipid accumulation in the cells was stained by ORO (A) and quantified (B). Results are presented as means ± SD and are from three independent experiments. ** *p* < 0.01.

## Data Availability

Included in article.
