# Supplementary material for: CYP1B1 Mediates Cigarette Smoke–Induced Lipid Accumulation in Alveolar Type 2 Cells
Source: FASEB J. 2025 Sep 19;39(18):e71062. doi: 10.1096/fj.202501439RR (PMC12448152; doi:10.1096/fj.202501439RR)
Supplement: Supplementary file 1 — Figure E1. EVE induces lipid accumulation in A549 cells. A549 cells were treated with 10% EVE for 3 days. Lipid droplets in A549 cells treated with 10% EVE were stained and quantified using ORO (A and B). (C) ORO staining was used to confirm the lipid accumulation after 3‐day CSE and EVE incubation (from 5%–15%). (D) The ORO staining was then quantified. Results are presented as means ± SD and are from three independent experiments. ns p > 0.05, * p < 0.05, ** p < 0.01. Figure E2. Inhibiting CYP1B1 suppresses EVE‐induced lipid accumulation in A549 cells. A549 cells were transfected with siCon or siCYP1B1 and cultured for 3 days. The lipid accumulation in the cells was stained by ORO (A) and quantified (B). Results are presented as means ± SD and are from three independent experiments. ** p < 0.01. [file FSB2-39-e71062-s001.docx]

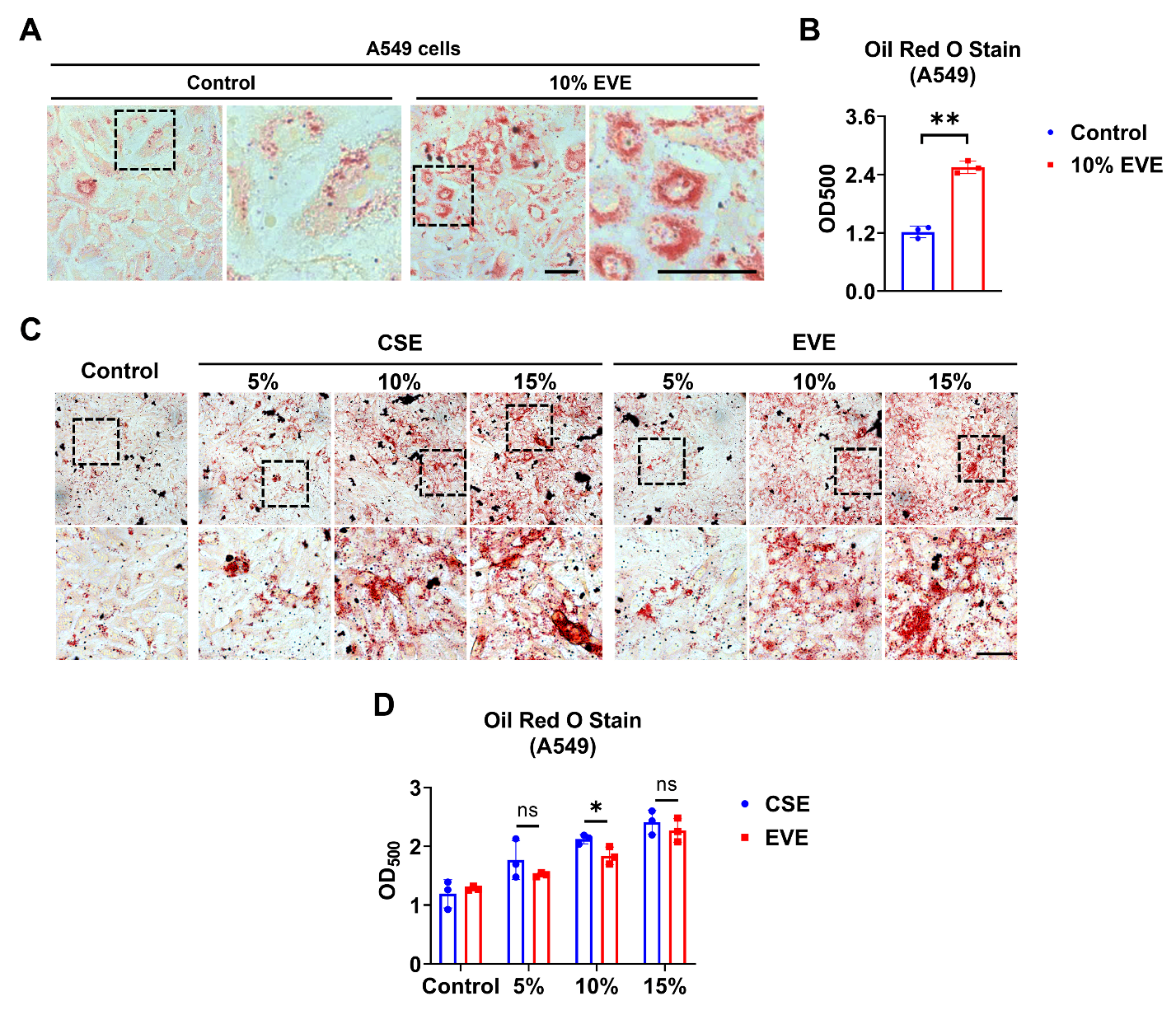


**Supplemental Figure Legend**

**Figure E1. EVE induces lipid accumulation in A549 cells.** A549 cells were treated with 10% EVE for 3 days. Lipid droplets in A549 cells treated with 10% EVE were stained and quantified using ORO (A and B). (C) ORO staining was used to confirm the lipid accumulation after 3-day CSE and EVE incubation (from 5-15%). (D) The ORO staining was then quantified. Results are presented as means ± SD and are from 3 independent experiments. ns *p* > 0.05, * *p* < 0.05, ** *p* < 0.01.


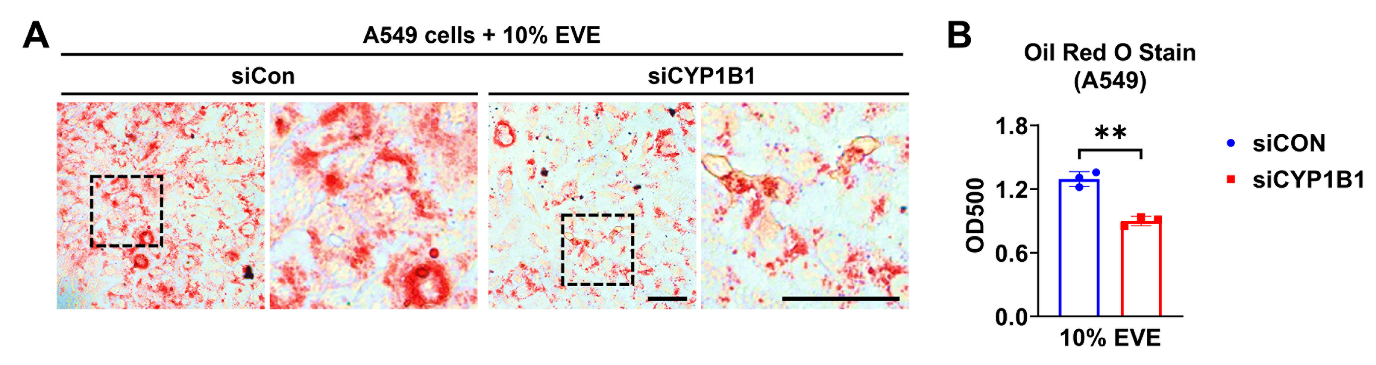


**Figure E2. Inhibiting CYP1B1 suppresses EVE-induced lipid accumulation in A549 cells.** A549 cells were transfected with siCon or siCYP1B1 and cultured for 3 days. The lipid accumulation in the cells was stained by ORO (A) and quantified (B). Results are presented as means ± SD and are from 3 independent experiments. ** *p* < 0.01.
